# Performance evaluation of non-ionic silicone surfactants OFX 0309 and DC 193C as a new approach in cloud point extraction – spectrophotometry for determination of atrazine in water samples†

**DOI:** 10.1039/c8ra00868j

**Published:** 2018-04-11

**Authors:** N. I. Mohd, M. Raoov, S. Mohamad, N. N. M. Zain

**Affiliations:** Integrative Medicine Cluster, Advanced Medical and Dental Institute (AMDI), Universiti Sains Malaysia 13200 Kepala Batas Penang Malaysia nurnadhirah@usm.my +60-04-562-2235; Department of Chemistry, Faculty of Science, Universiti Malaya 50603 Kuala Lumpur Malaysia

## Abstract

Cloud point extraction (CPE) – spectrophotometric method had been adopted for the separation of atrazine using the non-ionic silicone surfactant, polysiloxane polyether, as a new approach of atrazine extractor in CPE. There were two types of polysiloxane polyether (DC 193C and OFX 0309) studied to investigate the effectiveness of these surfactants toward atrazine in CPE method. Various effects of operating parameters such as the concentration of surfactant and atrazine, addition of salt and change of temperature and pH on the extraction of atrazine have been studied in detail to find the optimum conditions. The limit of detections (LODs) and quantifications (LOQs) were in the range of 0.09–0.59 μg L^−1^ and 0.31–1.95 μg L^−1^ in water samples, respectively. The method recoveries at two spiked levels were 84–105% (CPE-OFX 0309) and 59–69% (CPE-DC 193C) with good correlation of determination (*R*^2^) ranging from 0.9927–0.9993 for both methods. The Langmuir isotherm has been used for solubilisation study of surfactant and atrazine. The thermodynamic parameters have been determined such as Gibbs free energy (Δ*G*°) which increases with temperature, value of enthalpy (Δ*H*°) and value of entropy (Δ*S*°) which increase with the surfactant hydrophobicity.

## Abstract 2

The principle of cloud point extraction (CPE) adopted for the separation of atrazine.

## Graphical abstract

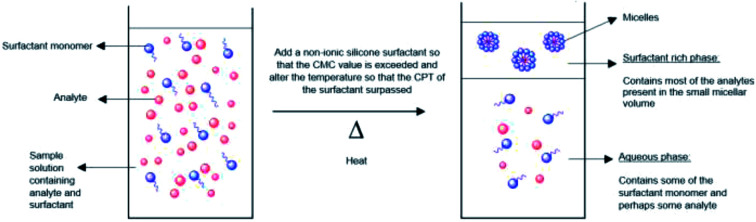

## 1. Introduction

Atrazine (2-chloro-4-(ethylamino-6-isopropylamino)-1,3,5-triazine) belongs to the chlorinated triazine groups which are the most heavily used pesticides in the maize crop industry. Although the use of atrazine is banned, the use of herbicides has been increasing due to the worldwide requirement for higher agricultural activities. Atrazine normally has a low adsorption in the soil where its migration easily takes place through the soil into the ground and surface waters.^1^ Atrazine can be transformed into degradation products which are even more toxic and persistent in the environment compared to the parent compounds.^2^ The existence of atrazine in the environment makes it transferable to animals and affects the food chain.^3^ The European Union (EU) legislation has established the maximum residue limit (MRL) for each individual herbicide to be set at 0.1 μg L^−1^ (ref. 4) but the United States Environmental Protection Agency (EPA) has set the maximum allowable level of atrazine at 50 μg L^−1^ in water samples.^5^ The excessive use of atrazine due to the increasing demand in agriculture industry caused severe contamination in the environment.^6^ The effects of atrazine towards the environment and its adverse effects on the human health cause people to pay attention in preventing its widespread. Therefore, sample pre-treatment process is necessary to remove any complex matrices in real sample before the analysis was carried out since atrazine exists in a low concentration in environment.^7^

Most of non-ionic surfactants in aqueous solutions possess the ability to decrease their solubility rapidly and become turbid when they are heated above a temperature called as cloud point temperature (CPT). The mechanism of separation is attributed to the rapid increase in the aggregation number of the surfactant's micelles due to the temperature elevation. The surfactant micelles are theoretically known to entrap several hydrophobic substances, isolating them from the bulk aqueous solution during their formation. An aqueous solution of a non-ionic surfactant gets separated into two phases above the CPT. The dilute phase contained the surfactant concentration slightly above the critical micelle concentration (CMC) and the other phase is surfactant rich phase, also known as coacervate phase which contained high concentration of solute and surfactant above the CMC. The solute that has been extracted in the small volume of the surfactant rich phase was then further subjected to spectrophotometry analysis.^8^ Among the advantages of using non-ionic surfactants are non-toxic, non-volatile and non-flammable which reduce hazard risk towards human and environment compared to volatile organic compounds (VOCs).^9^ Nowadays, Triton X series has been widely employed in CPE. However, aromatic chromophore in this surfactant has strong ultraviolet (UV) absorbance or fluorescence signals which becomes obstacles in UV and fluorescence detector. Therefore, non-ionic silicone surfactant, polysiloxane polyether (OFX 0309 and DC 193C) was used to overcome this problem because it has more flexible polysiloxane chains without any aromatic structure. Furthermore, it can form more compact micelle structures which offer low water content in the surfactant rich phase and also low in density; thus, enhancing the extraction efficiency.^10^ The non-ionic silicone surfactant are also used in pharmaceuticals and cosmetic products.^11^

Previously, the used of CPE technique in extraction of pollutant compounds from the environment had been developed and reported. CPE is one of the methods that attract attention of researcher owing to its various advantages such as relatively non-toxic surfactant used instead of toxic organic solvents, high extraction efficiency, modest energy consumption, lower cost and experimental convenient.^12^ The use of Triton series non-ionic surfactant in CPE method are the most widely reported article. Triton series had been reported to extract blue dye,^13^ crystal violet and methylene blue dye,^14^ phenol,^15^ polycyclic aromatic hydrocarbons (PAHs),^16^ phthalate esters^17^ and heterocyclic aromatic amine^18^ with the extraction efficiency up to 90%. Other than that, the use of Triton series in CPE method was also reported in extracting metallic compounds such as cadmium,^9^ lead,^19^ manganese,^20^ copper^21^ and uranium.^22^ The use of the non-ionic silicone surfactant, DC 193C was reported in extracting parabens with efficiency more than 80%.^23^ These authors concluded that the use of the non-ionic surfactants in CPE is a simple and effective process that eliminates the use of organic solvents from the liquid–liquid extraction process.^24^ Modern trends in analytical chemistry are moving towards simplifying steps and reducing the consumption of organic solvents, leading to the development of the CPE method.

The aim of present study was to investigate and develop the CPE – spectrophotometric method based on non-ionic silicone surfactants as a new approach for the extraction of atrazine compound in the water samples. The performance of these two types non–ionic silicone surfactant, OFX 0309 and DC 193C were investigated in detail towards atrazine using the CPE – spectrophotometric method. Non-ionic silicone surfactant was used as an extractor because it can form compact micelle structures, low water content and no aromatic structure that may become obstacles in UV spectrophotometry analysis. The effect of various operating parameters such as concentration of surfactant and atrazine, addition of salt, temperature and pH on the extraction efficiency of atrazine for both surfactants were studied to establish the optimum CPE conditions. Detailed studies on atrazine solubilisation and various thermodynamic parameters such as enthalpy (Δ*H*°), entropy (Δ*S*°), and Gibbs free energy (Δ*G*°) have been studied for both surfactants. This fundamental study will be helpful for future application for the extraction of organic pollutants or contaminants in water sample using non–ionic silicone surfactant in the CPE method.

## 2. Experimental

### 2.1 Chemicals and reagents

Used non–ionic silicone surfactant of OFX 0309 (3-(3-hydroxypropyl-heptatrimethylxyloxane); molecular weight: 5500 g mol^−1^) and DC 193C (PEG-12 dimethicone; molecular weight: 3100 g mol^−1^) were purchased from Ingredients Plus, Malaysia. Standard of atrazine (99% purity) (molecular weight: 215.68 g mol^−1^; *λ*_max_: 222 nm) was purchased from Dr Ehrenstorfer (Augsburg, Germany). The molecular structures were shown in Fig. SI1 (ESI†). All other chemicals used were of analytical grade and purchased from Sigma (St. Louis, MO, USA). Water used in the study was prepared from Sartorius Milli-Q water purification system (Aubagne, France). The standard stock solution of atrazine (1000 mg L^−1^) was prepared in methanol purchased from Friendemann Schmidt (Diviney Court Parkwood WA) and maintained at 4 °C protected from light. Meanwhile the working solutions were prepared daily by an appropriate dilution of the stock solutions with deionised water. Stock solution of OFX 0309 and DC 193C (20 w/v%) were prepared by dissolving 20 g in 100 mL volumetric flask with deionised water. Hydrochloric acid (HCl) and sodium hydroxide (NaOH) were used for the pH adjustment. Sodium sulfate (Na_2_SO_4_), potassium carbonate (K_2_CO_3_) and sodium carbonate (Na_2_CO_3_) were purchased from QRec, Malaysia were prepared by dissolving an appropriate amount in deionised water. For all experiments, both surfactants and atrazine compound were used without further purification.

### 2.2 Instrumentation

A Perkin Elmer Precisely, Model Lambda 25 UV-Vis spectrophotometer (Massachusettes, U.S.) with matched 10 mm quartz cells was used for spectral measurements of the atrazine. The parameters set for measurements were wavelength accuracy ± 0.5 nm, bandwidth 1.0 nm, and scan speed 400 nm min^−1^. A Memmert water bath (Schwabach, Germany) was used and maintained at the desired temperature. A Hanna Instrument pH meter (Rhode Island, USA) was used for the pH measurements.

### 2.3 Sample preparation

All the water samples were taken from selected area in Bertam, Penang, Malaysia. The tap water sample was taken from laboratory while the river water was taken from Sungai Lahar, Penang. The lake water was taken from the Vision Park, Penang while the pond water was taken from IPPT, USM. The paddy field water was taken from Kepala Batas, Penang. All the water samples were filtered using a 0.45 μm nylon membrane filter to remove any suspended particulate materials and stored at 4 °C before analysis.^25^ Appropriate amount of water samples spiked with different concentrations of atrazine were used to test the applicability of the method on real samples according to the method in section 2.4. All the spiked and unspiked (blank) water samples were analysed under the optimum procedure and the content of atrazine in the real water samples was then determined. The same procedure was applied for all water samples using CPE-DC 193C method.

### 2.4 Recommended cloud point extraction

In a typical CPE experiment, suitable aliquots containing a known concentration of atrazine (10 mg L^−1^) solution adjusted to pH 5 (using HCl or NaOH) was added to the OFX 0309 surfactant (1 mL of 0.4 v/v%) and 2.0 M of Na_2_SO_4_ (1 mL) in a capped glass centrifuge tube. The solution was mixed by a sonicator for 6 min. The solution was then kept in a thermostatic water bath at 50 °C for 15 min. After phase separation, the solution of the sample was then allowed to stand for 5 min at room temperature to achieve equilibrium. The appearance of two phases were observed, where the surfactant rich phase was at the top layer while the aqueous phase was at the bottom layer. The surfactant rich phase at the top layer could be separated by using a syringe which minimised the possibility of the cross-contamination of atrazine. Efficient spectrophotometer measurement can be obtained by separated out 0.5 mL of surfactant rich phase and diluted with 2 mL deionised water. The absorbance of the diluted surfactant rich phase was finally measured at 222 nm *vs.* reagent blank that was prepared in similar manner without spiking the atrazine analyte. In the CPE-DC 193C method, the same procedure was applied except for the concentration of Na_2_SO_4_. The concentration of 1.5 M Na_2_SO_4_ applied in the CPE-DC 193C method.

### 2.5 Solubilisation study

#### 2.5.1 Isotherm study

In the isotherm study, different concentrations of atrazine were studied in the range of 2.0–20.0 mg L^−1^. The initial pH of solution was adjusted by adding either HCl or NaOH to the pH 5 for both methods of CPE-OFX 0309 and CPE-DC 193C, whereas the other parameters such as the concentration of non-ionic silicone surfactants, concentration of salt, equilibration temperature and incubation time were remained constant. All the experimental data were collected in three replicates (*n* = 3) sample.

#### 2.5.2 Thermodynamic study

The effect of solution temperature on the solubilisation process of atrazine was conducted at different temperatures in the range of 30–80 °C, while the other parameters which are concentration of non-ionic silicone surfactants, concentration of salt, equilibration temperature and incubation time were remained constant. All the experimental data were collected in three replicates (*n* = 3) sample.

## 3. Results and discussions

### 3.1 Optimisation of the cloud point extraction conditions

The extraction efficiency of the CPE depends on the parameters such as the effect of pH, salt, temperature, concentration of atrazine, and surfactant. These parameters were investigated in detail to obtain the maximum extraction efficiency using two different non-ionic silicone surfactants. The extraction efficiency was defined as in eqn (1).^14^1
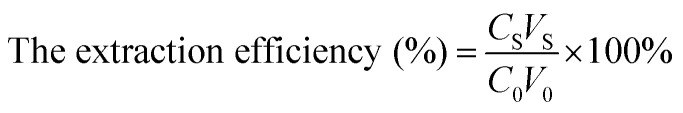
where *C*_S_ represents atrazine concentration in the surfactant rich phase volume, *V*_S_; while *C*_0_, represents the atrazine concentration in the initial sample-surfactant mixture of volume, *V*_0_.

#### 3.1.1 Effect of pH

**Fig. 1:**
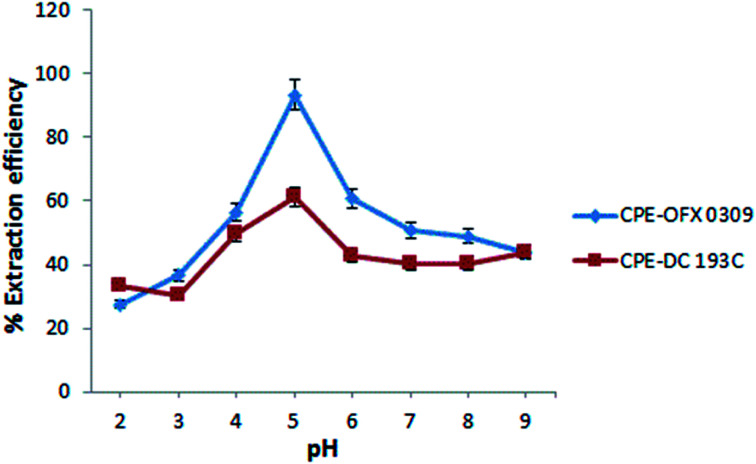
Effect of pH towards extraction efficiency of atrazine. Conditions: 10 mg L^−1^ of atrazine, 0.4 v/v% surfactant, 2.0 M of Na_2_SO_4_ at 50 °C.

In CPE, the pH of the sample solution plays an important role in the extraction of the target analytes because pH affects the analyte existing forms (ionic or neutral compound) during extraction process. Maximum extraction efficiency is achieved at the pH values where the target analyte prevails in neutral form due to non-dissociated type of non-ionic surfactant.^26^ In this work, the range of pH 2 until pH 9 was studied to determine the optimum pH. Fig. 1 shows the effect of pH on the extraction efficiency of atrazine (p*K*_a_ 1.7). The maximum extraction efficiency was obtained when the atrazine could exist as neutral form molecules (pH 5), which made the atrazine to be easily extracted.^27^ If the atrazine was too acidic, it would be too easily degraded and became protonated.^28^ As the pH was increased from pH 2 to pH 5, the extraction efficiency increased since atrazine would be distributed into surfactant rich phase as its solubility in water became low.^3^ The extraction efficiency started to drop at pH 6 and above because of atrazine (deprotonated) hydrolysed in alkali environment.^29^ Based on Fig. 1, both the CPE methods using OFX 0309 and DC 193C showed a similar trend in the effect of pH. However, the OFX 0309 surfactant shows better percentage of recovery. As the atrazine existed in molecular form at pH 5, the atrazine become more hydrophobic than ionic form due to the p*K*_a_ value of atrazine.^3^ Table SI1 (ESI†) shows the molecular structure of atrazine upon pH optimisation. Therefore, pH 5 was selected as optimum for both the CPE methods.

#### 3.1.2 Effects of salt

**Fig. 2:**
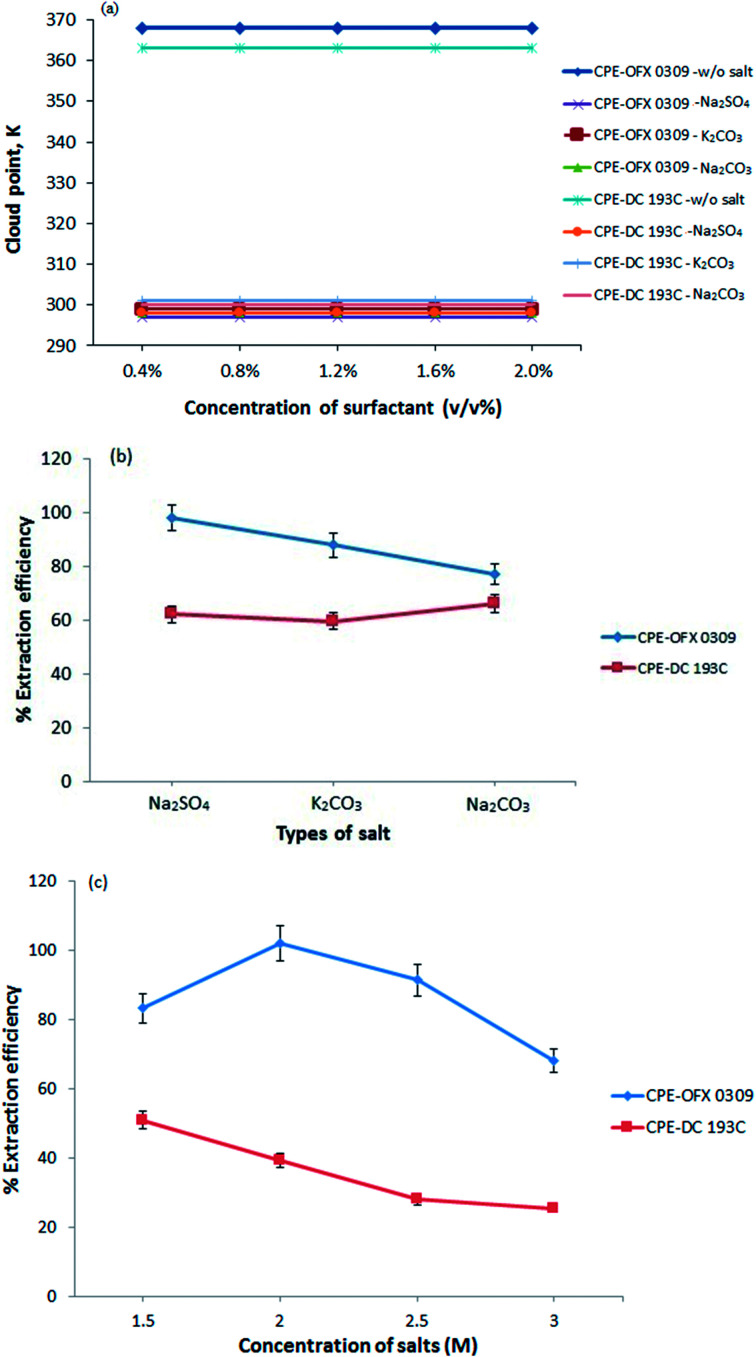
(a) Effect of salt towards the cloud point of OFX 0309 and DC 193C solutions. (b) Effect of types of salt towards extraction efficiency of atrazine. (c) Effect of salt concentration towards extraction efficiency of atrazine. Conditions: 10 mg L^−1^ of atrazine, 0.4 v/v% surfactant, pH 5 at 50 °C.

In CPE, the phase separation of the sample was done by heating the sample above CPT. However, the analyte that is sensitive to its volatility might lead to analyte losses due to high temperature applied.^8^ Thus, the salting out effect was introduced as an alternative in the phase separation due to its ability to reduce the solubility of atrazine in aqueous phase.^30^ The presence of salt is also known to decrease the CPT and increase the volume of the surfactant rich phase.^31^ This was demonstrated in Fig. 2(a) where by the present of salt, the CPT of the surfactants were reduced as they can form phases at room temperature. The use of low temperature may prevent the analyte degradation.^32^ The sample-surfactants solution required relative high temperature above the CPT of surfactants to form phases without the present of salt and this led to loss of analyte in sample solution.

In this work, there were three type of salts that showed a two phases separation include Na_2_SO_4_, K_2_CO_3_, and Na_2_CO_3_. This is due to the kosmotropic ions (CO_3_^2−^ and SO_4_^2−^) that exhibit a strong interaction with water molecule thus capable of breaking the water–water interaction. Fig. 2(b) shows that Na_2_SO_4_ had better extraction efficiency performances in CPE-OFX 0309 and CPE-DC 193C compared to the other two salts. The SO_4_^2−^ ion is likely to cause the decrease in the self-association of a water molecule by forcing more water goes into the aqueous phase and provide high extraction efficiency.^33^ The CO_3_^2−^ ion are not selected because the K_2_CO_3_ and Na_2_CO_3_ aqueous solution existed as basic solution (pH 11.35) which is not suitable for stability of atrazine and the addition of CO_3_^2−^ ions in the solution may interfere pH of sample solution.^34^ After comparing, Na_2_SO_4_ was chosen because the presence of Na^+^ cation that may reduce the CPT from the dehydration of polyethylene chain and since SO_4_^2−^ is a polyvalent ion, it will cause faster dehydration of water from the polyethylene chain.^35^ In addition, Na_2_SO_4_ salt increased the size of the micelles and aggregation number thus enhanced the solubility of atrazine molecules into the surfactant rich phase.^36^

The CPE extraction efficiency was also affected by the salt concentration. At optimum salt concentration, the water molecules in the surfactant rich phase goes into the aqueous phase. The different concentrations of salt were investigated ranging from 0.5 M to 3.0 M as shown in Fig. 2(c). However, at 0.5 M and 1.0 M, no phase separation was observed for both methods. Maximum extraction efficiency was observed at 2.0 M of Na_2_SO_4_ (CPE-OFX 0309) and 1.5 M of Na_2_SO_4_ (CPE-DC 193C). When the concentration is higher than 2.0 M in CPE-OFX 0309 and 1.5 M in CPE-DC 193C, the accuracy and reproducibility of the method were not in satisfactory due to difficulties in separating the extraction system into two phases. An optimum concentration of salt is required since it helps in reducing the concentration of “water free” in the surfactant rich phase and give maximum extraction efficiency.^37^ In each of the CPE methods, 2.0 M (CPE-OFX 0309) and 1.5 M (CPE-DC 193C) concentration of Na_2_SO_4_ were chosen for further study.

#### 3.1.3 Effects of temperature

**Fig. 3:**
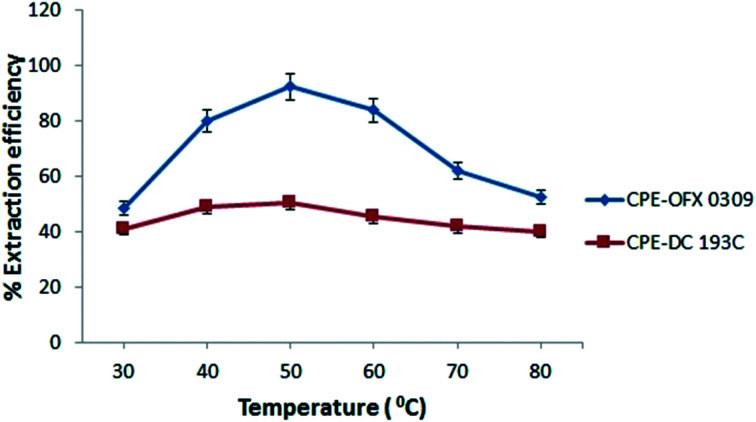
Effect of temperature towards extraction efficiency of atrazine. Conditions: 10 mg L^−1^ of atrazine, 0.4 v/v% of surfactant, pH 5, 2.0 M (CPE-OFX 0309); 1.5 M (CPE-DC 193C) of Na_2_SO_4_.

In both the CPE methods, the solubility of the atrazine molecules in aqueous phase reduces with the increase in temperature, thus, enhancing the extraction of atrazine in the surfactant rich phase. Based on this, temperature was used as a supportive parameter in enhancing the extraction efficiency of atrazine.^38^ The effects of temperature towards extraction efficiency were studied in the range of 30–80 °C. The extraction efficiency increased as the non-ionic surfactant became more hydrophobic with the increasing of temperature from 30 to 50 °C. Non-ionic surfactant appear moderately more hydrophobic at higher temperature due to an equilibrium shift that favoured dehydration of the ether oxygens, which leads to an increase in the number of concentration of micelles.^36^ Hence, the solubilisation capacity of the micellar solution increases with temperature leading to an increase in the extraction of atrazine.^39^ At a temperature above CPT, the atrazine compound will be solubilised into the surfactant rich phase.^40^ Thus, by reducing the CPT value due the present of Na_2_SO_4_, low temperature would be applicable in CPE procedure and provide maximum extraction efficiency. Based on Fig. 3, the extraction efficiency started to decrease at 60 °C due to the excessive high temperature which lead to the decomposition of atrazine.^41^ Therefore, 50 °C was chosen as the optimum condition in both CPE methods.

#### 3.1.4 Effects of surfactant concentration

**Fig. 4:**
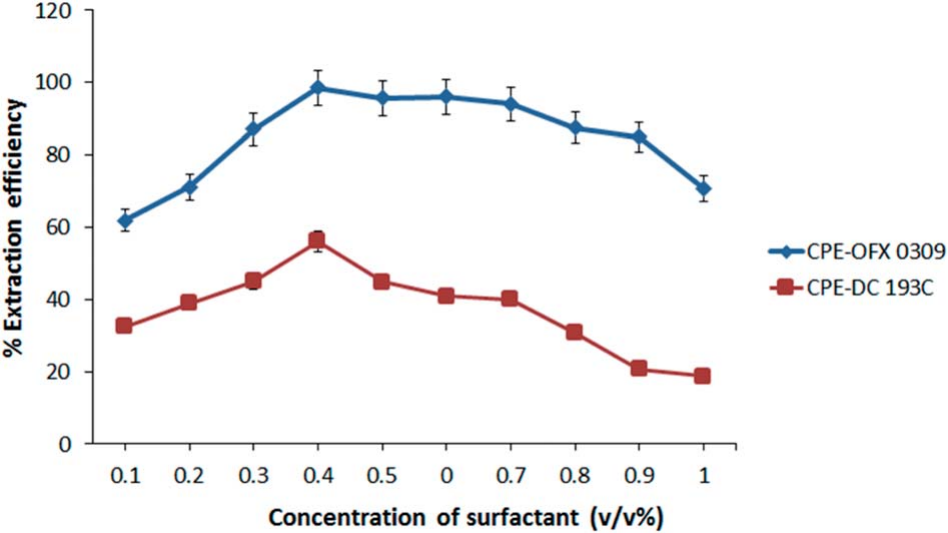
Effect of surfactant concentration towards extraction efficiency of atrazine. Conditions: 10 mg L^−1^ of atrazine, 2.0 M of Na_2_SO_4_ at 50 °C.

Increase of surfactant concentration usually increases the volume of surfactant rich phase to maintain the material balance. Other than that, the extraction of atrazine into the surfactant rich phase also enhanced along the surfactant concentration. However, the concentration of surfactant must be sufficient for the formation of micelle aggregates and quantitative extraction of the target analytes. The increase in surfactant concentration will increase the extraction efficiency and also the concentration of the micelles in the solution, resulting in more atrazine solubilisation in the micelles.^42^ The effects of surfactant concentration on the extraction efficiency were evaluated in the range of 0.1–1.0 v/v% as shown in Fig. 4. The extraction efficiency increased as the concentration was increased up to 0.4 v/v% and decreased as the concentration was increased up to 1.0 v/v%. This result might be related to the presence of the high amount of surfactant, which results in an increase in the viscosity of the surfactant rich phase ^43^and lead to a low extraction of atrazine.^44^ At the lower concentration of surfactant (<0.4 v/v%), the solubilisation efficiency of the atrazine was low probably due to assemblies of surfactants that were inadequate to quantitatively entrap the hydrophobic species.^45^ Therefore, 0.4 v/v% was selected for further study in CPE-OFX 0309 and CPE-DC 193C methods.

#### 3.1.5 Effects of atrazine concentration

**Fig. 5:**
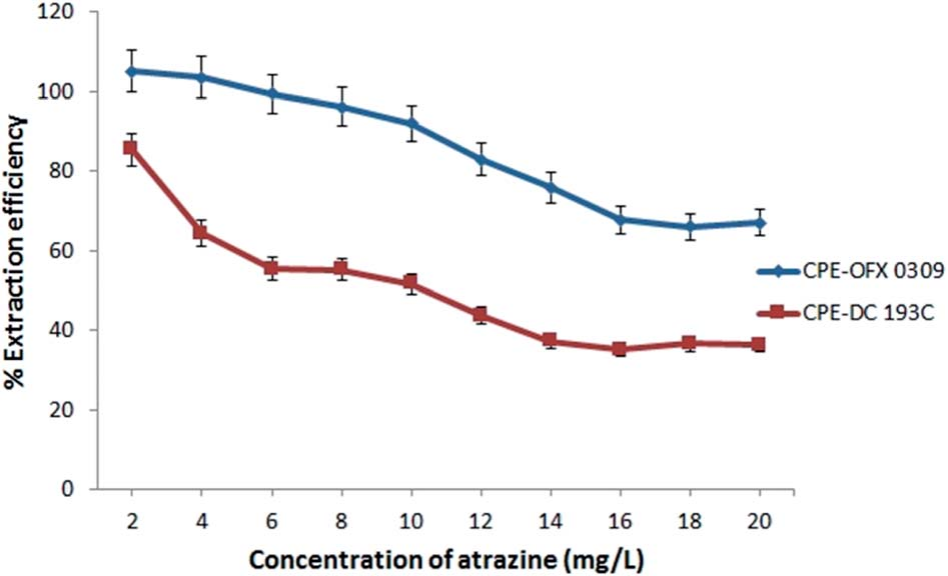
Effect of atrazine concentration towards extraction efficiency of atrazine. Conditions: 0.4 v/v% surfactant, pH 5, 2.0 M (CPE-OFX 0309); 1.5 M (CPE-DC 193C) of Na_2_SO_4_ at 50 °C.

The solubilisation study was conducted to investigate the effects of atrazine concentration ranging from 2.0–20.0 mg L^−1^ towards the capability of surfactant at a constant concentration of the surfactant for both the CPE methods. Fig. 5 shows that by increasing the atrazine concentration, the extraction efficiency was reduced from 105 to 67% in the CPE-OFX 0309 method. Meanwhile, in the CPE-DC 193C method, the extraction efficiency was reduced from 85 to 36%. At a lower concentration of atrazine, a large number of vacant surface sites of surfactant existed and was available for the extraction.^46^ However, by increasing the atrazine concentration, the remaining vacant surface sites became difficult to be occupied due to the repulsive force between the atrazine molecules on the surfactant and bulk phase.^47^ This is also due to the increasing number of atrazine molecules competing for available active sites.^48^ In the CPE-OFX 0309 method, the OFX 0309 surfactant provided the higher extraction efficiency of atrazine as compared to the DC 193C surfactant due to more vacant sites for the atrazine molecules to be solubilised in OFX 0309 surfactant.

### 3.2 Solubilisation isotherm

#### 3.2.1 Isotherm study

Distribution of various concentration of atrazine between surfactant rich phase and diluted phase at equilibrium can be studied by the solubilisation of the atrazine into the interior or outer layer of micelles. Thus, by assuming a homogeneous monolayer, isotherm study was conducted to understand the mechanism of atrazine solubilisation.^49^ In the isotherm study, Langmuir has been the most widely used model to describe the sorption of solute from water samples.^50^ The Langmuir theory explains the solubilisation process that takes place at the specific site on the surface of sorbent until maximum solubilisation and no further solubilisation will take place when the site was occupied with solute.^51^ The solubilisation capacity (*m*) and energy of solubilisation (*n*) in the two CPE methods, CPE-OFX 0309 and CPE-DC 193C, were determined through the calculation of data. The Langmuir isotherm had been used to explain the solubilisation of both surfactants with atrazine due to its success in describing the solubilisation processes. Eqn (2) gives the expression of the Langmuir isotherm model:2
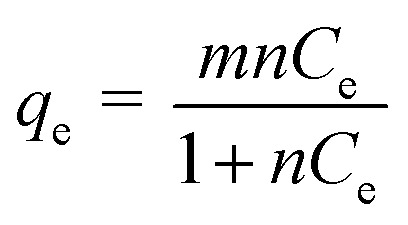
where *q*_e_ is the moles of atrazine solubilised per mole of the surfactant at equilibrium (mol mol^−1^). *C*_e_ is the equilibrium concentration of atrazine in the diluted phase (mol L^−1^). The constants *m* and *n* are the Langmuir constants where *m* signifies the solubilisation capacity (mol mol^−1^) and *n* is related to the energy of solubilisation (L mol^−1^).^52^

**Fig. 6:**
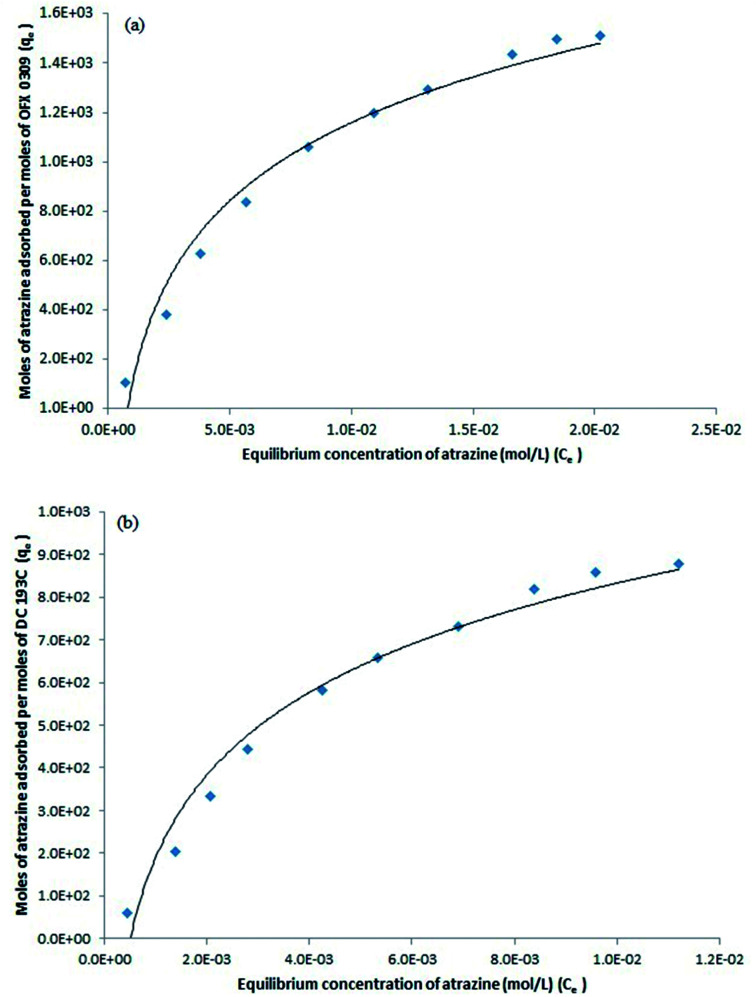
Solubilisation isotherm of atrazine over surfactants (a) OFX 0309 (b) DC 193C in CPE.

The study was conducted by varying the initial concentration of atrazine in the range of 2.0–20.0 mg L^−1^ and all the experiments were carried out at 50 °C. After phase separation, the volume of surfactant rich phase and diluted phase were recorded while the concentration of atrazine in the surfactant rich phase and diluted phase was analysed using spectrophotometer. Fig. 6(a) and (b) shows the solubilisation isotherm in the CPE-OFX 0309 and CPE-DC 193C methods illustrated by plotting *q*_e_*vs. C*_e_, respectively.

#### 3.2.2 Evaluating the values of *m* and *n*

The Langmuir equation can be linearized into the eqn (3)3
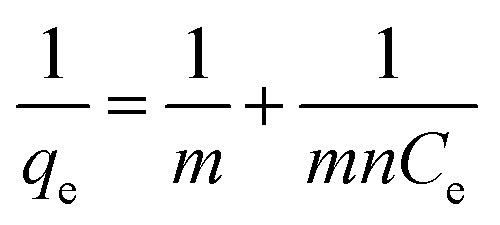


Based on the eqn (3), a plot of 1/*q*_e_*vs.* 1/*C*_e_ gives a straight line with slope 1/*mn* and intercepts 1/*m*. The slope and intercept of the linear form of the Langmuir isotherm model were used to determine the values of *m* and *n*. It was reported that when the diluted phase in CPE was separated, the solubilisation capacity (*m*) and energy of solubilisation (*n*) were constant. The values of *m* and *n* in the both CPE-OFX 0309 and CPE-DC 193C methods were tabulated in Table 1.

**Table 1:** Calculated *m* and *n* in both of the CPE methods

Parameters	CPE-OFX 0309	CPE-DC 193C
*m* (mol mol^−1^)	2 × 10^−4^	5 × 10^−5^
*n* (L mol^−1^)	7.14 × 10^8^	5 × 10^9^
*R* ^2^	0.9956	0.9946

**Fig. 7:**
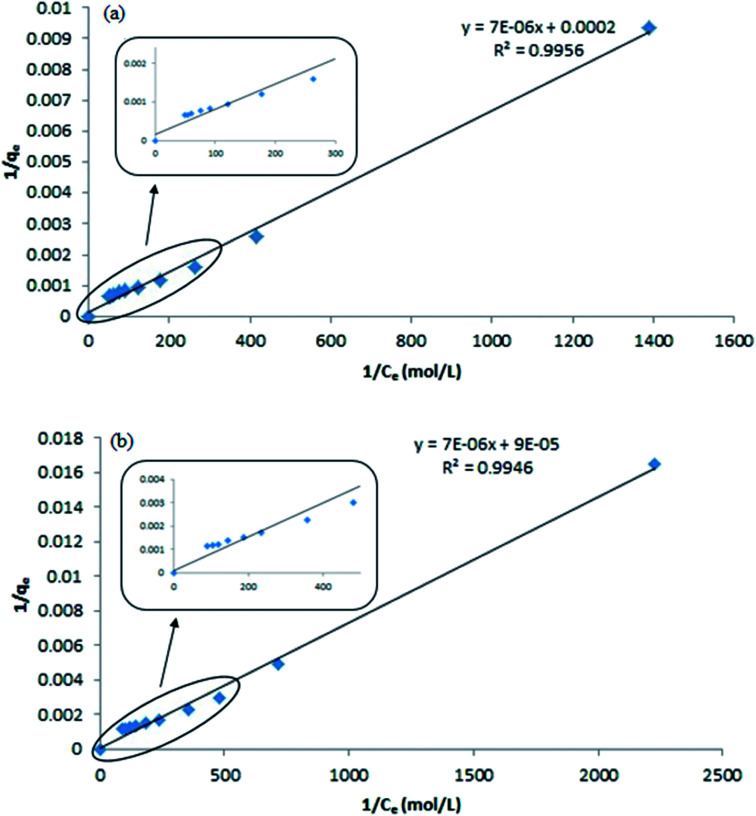
Plotting 1/*q*_e_*vs.* 1/*C*_e_ for *m* and *n* calculation. (a) CPE-OFX 0309 (b) CPE-DC 193C.

**Fig. 8:**
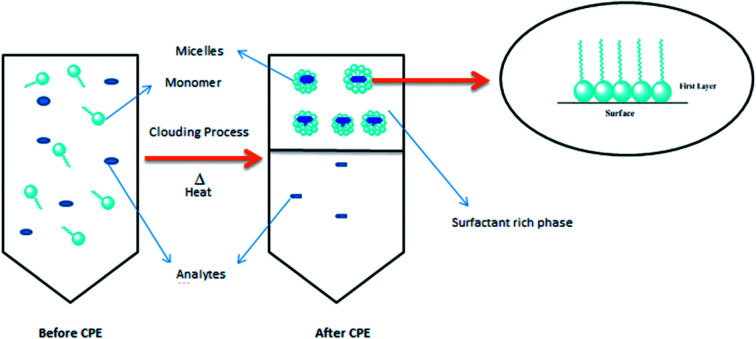
Schematic diagram of CPE methods and proposed Langmuir monolayer model.

From the plot of 1/*q*_e_*vs.* 1/*C*_e_ in the CPE-OFX 0309 and CPE-DC 193C methods, the correlation of determination obtained was greater than 0.89 which were 0.9956 in CPE-OFX 0309 and 0.9946 in CPE-DC 193C methods. The data obtained were as shown in Fig. 7(a) and (b). The solubilisation was confirmed to be the Langmuir model as the value of correlation of determination was greater than 0.89.^53^ The isotherm study of the CPE-OFX 0309 and CPE-DC 193C methods was fitted well with Langmuir isotherm. The monolayer coverage was proposed to be suitable according to the Langmuir model as shown in Fig. 8.

From the calculated values of the solubilisation capacity (*m*) and energy of solubilisation (*n*) in Table 1, the value of solubilisation capacity (*m*) in CPE-OFX 0309 was 2 × 10^−4^ mol mol^−1^ while in CPE-DC 193C, the value was 5 × 10^−5^ mol mol^−1^. The value of *m* obviously increased along with the increases of the surfactant hydrophobicity. Based on the value of *m*, it showed that the chain length of surfactant was known to affect its hydrophobicity.^54^ The longer the hydrophobic chain due to the increasing molecular weight may reduce water solubility of surfactant thus increases surfactant aggregation in solution.^55^ The solubilisation of the atrazine in surfactant was balanced by its tail and head. The hydrophobic tail of the surfactant molecule tends to be attracted to the atrazine, whereas the hydrophilic head has higher affinity toward water.^56^ However, the hydrophilic head was interrupted by the addition of salt which leads to the higher solubilisation of analytes in the surfactant rich phase.^12^ From the calculated values, it was suggested that the OFX 0309 surfactant would be effective as the surfactant in the extraction of atrazine as compared to DC 193C surfactant.

The energy value of solubilisation (*n*) showed that less energy was required by OFX 0309 surfactant (7.14 × 10^8^ L mol^−1^) in CPE-OFX 0309 method compared to the DC 193C surfactant in CPE-DC 193C method (5 × 10^9^ L mol^−1^) for the solubilisation of atrazine into the surfactant micelles. It can be concluded that the strong binding interaction between atrazine and the surfactant led to the less energy required to solubilise atrazine into the surfactant rich phase. Thus, making the OFX 0309 surfactant as a better extractor of atrazine compound in CPE. In both the CPE methods, the proposed mechanism interaction might involve the hydrogen bonding interaction where the hydrogen bonding act as the impulse in the surfactant solubilisation as shown in Fig. SI2 (ESI†).^57^

### 3.3 Determination of thermodynamic parameters

The change in the values of free energy (Δ*G*°), entropy (Δ*S*°) and enthalpy (Δ*H*°), were affected by the temperature. It was suggested that increasing the temperature reduces the interaction between hydrophilic part of the surfactant and water thus, the dehydration of water occurred in the external layer of micelles.^58^ The influence of temperature was studied in the range of 30–80 °C. The thermodynamic parameters of Δ*G*°, Δ*S*°, and Δ*H*° for this extraction process was determined using the following equations:

4 Δ*G*° = Δ*H*° − *T*Δ*S*°

5
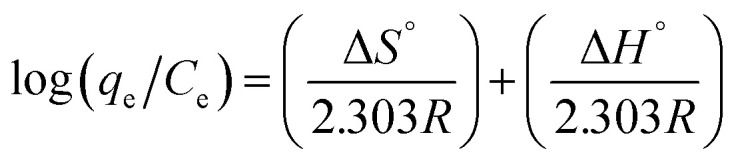
6
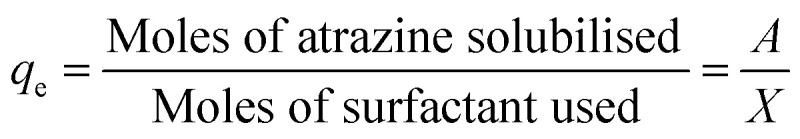


Moles of the solubilized atrazine can be obtained through the mass balance;

7 *A* = *V*_0_*C*_0_ – *V*_d_*C*_e_

8 *X* = *C*_s_*V*_0_

where *q*_e_ is the moles of solubilised atrazine per moles of surfactant used, *C*_e_ is the equilibrium concentration of atrazine (mol L^−1^) after the completion of two phases, and *T* is the temperature in Kelvin. *q*_e_/*C*_e_ is known as the solubilisation affinity while *A* is the mole of solubilised atrazine into the surfactants. *V*_0_ is the volume of initial solution before the CPE, *V*_d_ is the volume of aqueous phase after the CPE. *C*_0_ is the atrazine concentration in initial solution and *C*_s_ is the concentration of the atrazine in the surfactant rich phase. The thermodynamic parameters Δ*G*°, Δ*H*°, and Δ*S*° were noted to be in the linear range of *q*_e_*vs. C*_e_ plot, which can be calculated using the experimental data in both the CPE methods. Referring to eqn (4), the values of Δ*G*° can be calculated by knowing the Δ*H*° and Δ*S*°. Eqn (5) was used to calculate Δ*H*° and Δ*S*° that were obtained from a plot of log(*q*_e_/*C*_e_) *vs.* 1/*T*.^59^ The values of Δ*G*°, Δ*H*°, and Δ*S*° for the both methods were calculated and tabulated in Table 2.

**Table 2:** Thermodynamic parameters in both of the CPE method at different temperature

Methods	Temperature (K)	−Δ*G*° (J mol^−1^), (×10^4^)	Δ*H*° (J mol^−1^)	Δ*S*° (J mol^−1^), (×10^2^)
CPE-OFX 0309	303	34.44	11.80	11.37
	313	35.58		
	323	36.72		
	333	37.85		
	343	38.99		
	353	40.13		
CPE-DC 193C	303	24.18	10.35	7.98
	313	24.98		
	323	25.79		
	333	26.58		
	343	27.37		
	353	28.17		

#### 3.3.1 Variation of Gibbs-free-energy (Δ*G*°) during CPE of atrazine

**Fig. 9:**
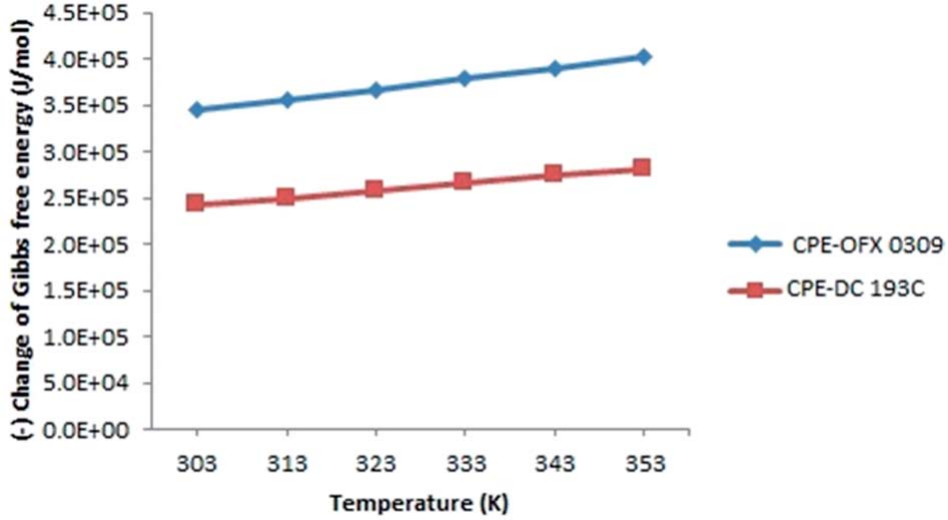
Variation of Gibbs free energy (Δ*G*°) along temperature at constant concentration of surfactants and atrazine.

The variations of Δ*G*° with different temperatures at the constant atrazine concentration for both the CPE methods are shown in Fig. 9. It was noticed that the solubilisation ability increased linearly upon the increment of the temperature.

The negative value of Δ*G*° indicates that the atrazine solubilisation process was spontaneous and thermodynamically favourable.^60^ The increment of the negative values of Δ*G*° on elevating temperature showed the driving force of solubilisation as signified from the increases in atrazine extraction on increasing temperature. This phenomenon can be explained by the hydrophobicity of the surfactant where OFX 0309 was more hydrophobic than DC 193C surfactant. The slight shifting of Δ*G*° value between OFX 0309 and DC 193C surfactant with the increasing temperature indicates that the micellization was driven by the hydrophobicity of the surfactant.^61^

The change in the enthalpy (Δ*H*°) is equal to the sum of the heat supplied during CPE. The positive values of Δ*H*° 11.80 J mol^−1^ in CPE-OFX 0309 and 10.35 J mol^−1^ in CPE-DC 193C reflects to the endothermic natural of solubilisation affinity of atrazine in the micellar phases.^62^ The endothermic nature was also shown by the increase in the amount of solubilisation with temperatures due to the increase number of hydrophobic micelles in surfactant rich phase while the CMC of the non-ionic surfactants decrease on increasing temperature.^63^ Increasing the temperature was known to stimulate the rate of diffusion of atrazine in the external surface of surfactant and decrease the viscosity of the solution.^47^ Value of Δ*H*° in CPE-OFX 0309 method was higher than CPE-DC 193C method due to the high rate of solubilisation of atrazine with OFX 0309 surfactant. This is because the OFX 0309 surfactant has a high hydrophobicity towards atrazine. The effects of temperature can be explained based on the hydrogen bonding. In the water samples, the hydrogen bonds were broken at the higher temperature, causing the atrazine to solubilise into surfactant. This solubilisation process involved the required of heat.^64^

Meanwhile, entropy is mainly depending on the free surfactant molecules since the surfactant concentration is much higher than the atrazine molecules in the solution. The positive value of Δ*S*° corresponded to a decrease in the degree of freedom of the solubilisation atrazine, which showed the good affinity and organization of the atrazine molecules in a random fashion of micellar.^65^ The CPE-OFX 0309 method showed the higher value of Δ*S*° which was 11.37 J mol^−1^ as compared to the CPE-DC 193C method at 7.98 J mol^−1^. From the obtained Δ*S*° value, the use of OFX 0309 surfactant in the CPE provided less randomness of the atrazine and surfactant. This causes more atrazine to be attracted to OFX 0309 surfactant than the DC 193C surfactant.^66^ Based on this, with the used of more hydrophobic surfactant, positive values of Δ*H*° and Δ*S*° were observed, where the values indicated the OFX 0309 surfactant as better extractant towards atrazine than the DC 193C surfactant due to their different hydrophobicity.

### 3.4 Application of water samples

#### 3.4.1 Analytical performance of the methods

The analytical figures of merit of the proposal methodology were summarized in Table 3. The calibration curve of each water samples was constructed in the concentration range of 1–2000 μg L^−1^ for the CPE-OFX 0309 method and 3–2000 μg L^−1^ in the CPE-DC 193C method. The calibration curves of the real water samples was constructed individually on the water samples to reduce the effect sample matrices, the calibration curves constructed showed a satisfactory linearity with correlation of determination (*R*^2^) in the range of 0.9974–0.9993 (CPE-OFX 0309) and 0.9927–0.9985 (CPE-DC 193C). The limit of detections (LODs) and limit of quantifications (LOQs) were calculated using 3*s*/*b* and 10*s*/*b*, respectively, where *s* is the standard deviation of ten replicate measurements of blank water sample and *b* is the slope of calibration curve. The LODs calculated were 0.09–0.59 μg L^−1^ and the LOQs were 0.31–1.95 μg L^−1^. The percentage relative standard deviation (% RSD) obtained were in range of 3.09–7.93% for the CPE-OFX 0309 method and 2.61–8.12% for the CPE-DC 193C method, respectively.

**Table 3:** Analytical parameters of the CPE – spectrophotometric method^a^

Methods	Sample	Linear Equation	*R* ^2^	% RSD, *n* = 3	LOD (μg L^−1^)	LOQ (μg L^−1^)
CPE-OFX 0309	1	*y* = 0.027*x* + 0.103	0.9974	0.75	0.12	0.40
	2	*y* = 0.035*x* + 0.018	0.9986	1.28	0.09	0.31
	3	*y* = 0.016*x* + 0.071	0.9982	0.93	0.21	0.69
	4	*y* = 0.011*x* + 0.109	0.9974	0.29	0.31	1.03
	5	*y* = 0.016*x* + 0.212	0.9993	0.14	0.20	0.67
CPE-DC 193C	1	*y* = 0.017*x* + 0.188	0.9927	0.23	0.18	0.61
	2	*y* = 0.014*x* + 0.197	0.9942	1.14	0.24	0.81
	3	*y* = 0.013*x* + 0.213	0.9982	1.73	0.26	0.85
	4	*y* = 0.036*x* + 0.309	0.9946	0.24	0.59	1.95
	5	*y* = 0.008*x* + 0.341	0.9985	0.28	0.42	1.40

a (1) = Tap water; (2) = River water; (3) = Lake water; (4) = Pond water; (5) = Paddy field water.

#### 3.4.2 Application of proposed CPE methods to water samples

In order to validate the proposed method in the determination of carcinogenic atrazine in the water samples, recovery study was conducted by spiking the samples. Two different concentration levels which are 1 and 1000 μg L^−1^ in the CPE-OFX 0309 method, 5 and 1000 μg L^−1^ in the CPE-DC 193C method were analysed. Each spiked concentration level was analysed in three replicates of samples (*n* = 3). Satisfactory accuracy of the recovery study was observed, and the average recovery were tabulated in Table 4.

**Table 4:** Analysis of atrazine in water samples (*n* = 3) by the developed method of CPE – spectrophotometry^a^

Samples	CPE-OFX 0309			CPE-DC 193C		
	Added (μg L^−1^)	bFound	Recovery (%)	Added (μg L^−1^)	bFound	Recovery (%)
Tap water	0	nd	—	0	nd	—
	1	0.11 ± 0.007	98	5	0.07 ± 0.005	69
	1000	0.13 ± 0.006	100	1000	0.11 ± 0.006	68
River water	0	nd	—	0	nd	—
	1	0.086 ± 0.002	91	5	0.03 ± 0.001	64
	1000	0.10 ± 0.004	84	1000	0.08 ± 0.004	69
Lake water	0	nd	—	0	nd	—
	1	0.08 ± 0.006	92	5	0.03 ± 0.001	65
	1000	0.10 ± 0.003	98	1000	0.04 ± 0.002	69
Pond water	0	nd	—	0	nd	—
	1	0.15 ± 0.008	105	5	0.13 ± 0.001	60
	1000	0.18 ± 0.001	98	1000	0.09 ± 0.005	63
Paddy field water	0	nd	—	0	nd	—
	1	0.23 ± 0.008	88	5	0.08 ± 0.007	59
	1000	0.21 ± 0.01	85	1000	0.09 ± 0.006	63

a nd = not detected.

b Mean ± standard deviation.

## 4. Conclusions

In the present study, CPE – spectrophotometric method based on non-ionic silicone surfactant of OFX 0309 and DC 193C as a new approach was successfully applied to extract carcinogenic atrazine in water samples. The effect of concentration of surfactant and atrazine, salt, temperature, and pH were investigated in CPE method. The performance of OFX 0309 surfactant was found to be better than DC 193C surfactant in extracting atrazine using CPE method. This is because of OFX 0309 surfactant has higher molecular weight which contribute to higher hydrophobicity of surfactant. The use of CPE methods offers several benefits such as low cost, safer, and good extraction performance towards atrazine compound. A Langmuir type isotherm, where monolayer coverage of atrazine onto the surfactant was effectively describes the solubilisation isotherm of atrazine with non-ionic silicone surfactant, OFX 0309 and DC 193C. In the thermodynamic analysis, the detailed study revealed that the changes in Gibbs free energy (Δ*G*°) increased with temperature while the changes in enthalpy (Δ*H*°) and entropy (Δ*S*°) increased with the surfactant hydrophobicity. Both CPE methods were found to be endothermic and spontaneous in nature. The positive values of Δ*S*° indicate that the solubilised atrazine is organized in randomness fashion of surfactant rich phase. The use of both non-ionic silicone surfactants of OFX 0309 and DC 193C are compatible to UV-Vis spectrophotometer and the extraction performance of the surfactants towards atrazine are based on the surfactant hydrophobicity. The non-ionic silicone surfactant not only suitable to be used as extractor of organic pollutant but also safe for human consumption.

## Conflicts of interest

The authors have declared that no conflict of interest.

## Supplementary Material

RA-008-C8RA00868J-s001
