# Supplementary material for: Performance evaluation of non-ionic silicone surfactants OFX 0309 and DC 193C as a new approach in cloud point extraction – spectrophotometry for determination of atrazine in water samples
Source: RSC Adv. 2018 Apr 11;8(24):13556–66. doi: 10.1039/c8ra00868j (PMC9079888; doi:10.1039/c8ra00868j)
Supplement: RA-008-C8RA00868J-s001 [file RA-008-C8RA00868J-s001.pdf]

Electronic Supplementary Material (ESI) for RSC Advances.
This journal is © The Royal Society of Chemistry 2018

List of Supporting Information

Fig. SI1: Molecular structure of (a) DC 193C (b) OFX 0309 (c) atrazine**.**

Fig. SI2: Proposed interaction between the surfactants (OFX 0309 and DC 193C) and atrazine.

Table SI1: Molecular structure of atrazine in the pH condition.

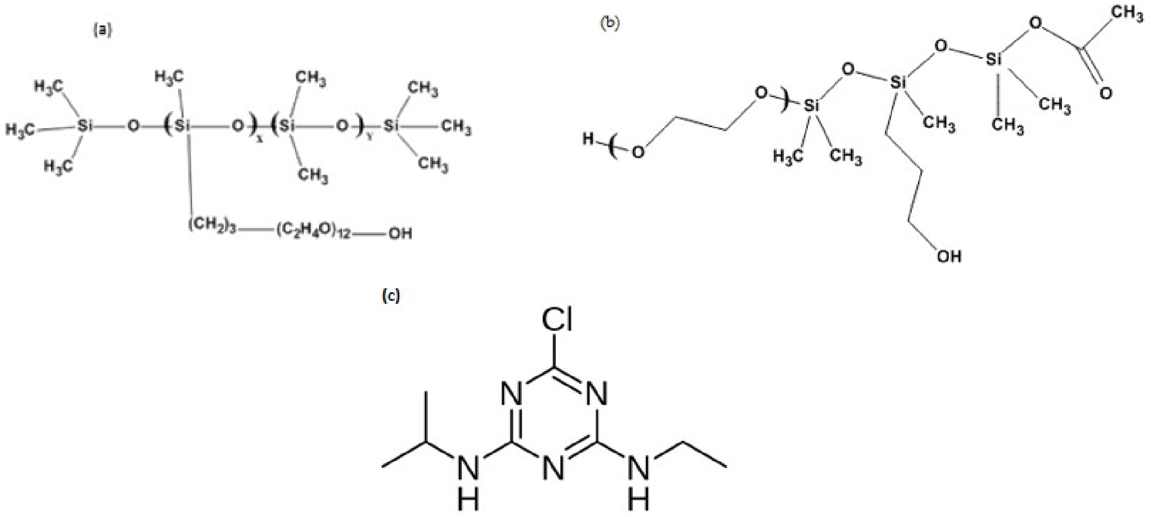

Fig. SI1: Molecular structure of (a) DC 193C (b) OFX 0309 (c) atrazine**.**

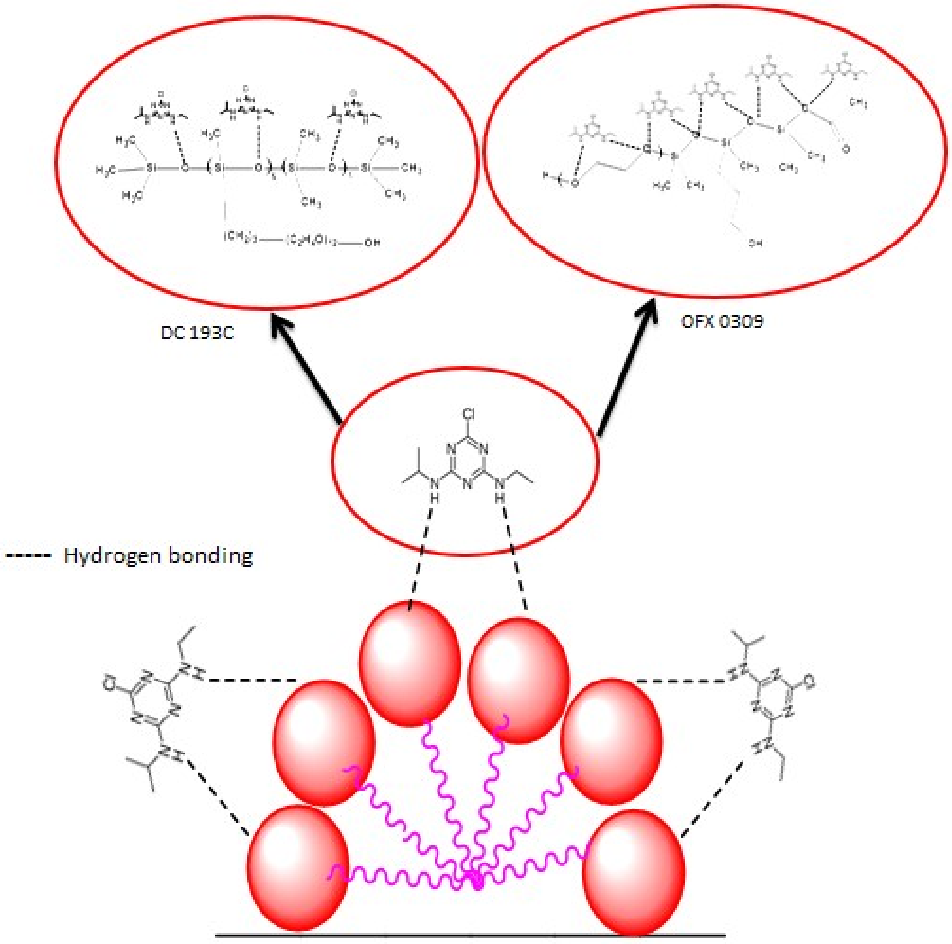

Fig. SI2: Proposed interaction between the surfactants (OFX 0309 and DC 193C) and at

Table SI1
Molecular structure of atrazine in the pH condition.

	Protonated<br>(pH < 5)	Neutral<br>(pH = 5)	Deprotonated<br>(pH > 5)
**Atrazine**	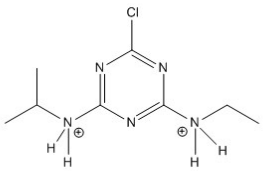	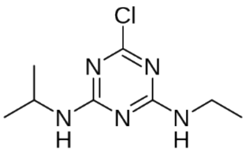	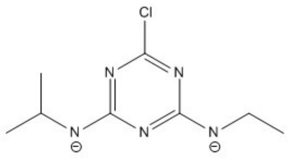
